# Experimental Investigation on Part Quality and Dust Emission during Minimum Quantity Lubricated (MQL) Edge Finishing of Granite

**DOI:** 10.3390/mi13101714

**Published:** 2022-10-11

**Authors:** Haithem Bahri, Victor Songmene, Jules Kouam

**Affiliations:** Department of Mechanical Engineering, École de Technologie Supérieure (ÉTS), Montreal, QC H3C 1K3, Canada

**Keywords:** grinding, granite, edge-finishing, MQL, fine particles (FPs), surface finish

## Abstract

Edge-finishing of granites by grinding is a process frequently used in the granite processing industry to generate the final desired shape and edge quality of products. However, this process releases significant amounts of fine and ultrafine particles (FPs and UFPs) containing crystalline silica. When inhaled, this dust can cause silicosis disease and threaten the health and safety of workers. The purpose of this study is to optimize the process by decreasing the concentrations of dust generated while also maintaining the required surface finish. Experimental tests were planned and performed on granite samples using a full factorial design. Two cutting tool edge shapes were studied (chamfer and concave) using G150 and G600 grit size tools, at various spindle speeds (1500, 2500, 3500 rpm), feed rates (500, 1000, 1500 mm/min) and lubrication flow rates (20, 40, 60 mL/min). The findings show that the particle emissions as well as the surface finish depend on the tool shape, its grit size, and the machining and lubrication parameters used. Higher MQL flow rates led to better finished surface quality and lower concentrations of fine dust. Polishing with flood lubrication reduces the maximum number concentration of FPs corresponding to particles smaller than 1 µm diameter by about 85% as compared to dry polishing and produced the best surface finish. Polishing with lubrication in MQL mode at 60 mL/min led to the production of part with Ra-value comparable with that obtained in flood lubrication condition.

## 1. Introduction

Over the last 30 years, the use of granite in modern construction and kitchen countertops has increased year after year. In Quebec, the annual production of raw granite increased from 67,000 tons in 1991 to 175,000 tons in 2000, which represents an average annual increase of 11% [1]. Granite is a material that contains mainly quartz, feldspar and micas such as biotite or muscovite. As the percentage of quartz varies from 2% to 60% according to the type of granite [2], its processing can generate fine particles (FPs) and ultra fine particles (UFPs) of crystalline silica (SiO_2_) that constitute a health hazard for workers. According to Hinds (1999) [3], UFPs are particles with a nanometric size ranging from 1 to 100 nm in diameter, and FPs are particles with a micrometric size and a diameter of between 1 and 10 μm. Quartz crystals are classified as a human carcinogen by the IARC (International Agency for Research on Cancer, Lyon, France) and they are linked to lung cancer [4]. Several studies have shown that prolonged exposure to high levels of these crystals causes serious lung and respiratory diseases such as chronic bronchitis and silicosis [5,6,7]. According to the INSPQ (Institut National de Santé Publique du Québec, Canada), 360 new cases of silicosis, all of occupational origin, were recorded between 2006 and 2017 [8]. In order to tackle this hazard, several pieces of legislation have been created all over the world to regulate the concentrations of crystalline silica dust in indoor air, especially the inhalable fraction with diameter less than 50 µm. In Spain, the environmental limit value for daily exposure to inhalable crystalline silica dust is lower than 0.1 mg/m^3^ [9]. In Quebec, RSST (Réglement sur la Santé et la Sécurité du Travail, Quebec, Canada) has set a permissible exposure limit (PEL) for quartz that must not exceed 0.1 mg/m^3^ in 8 h TWA (Time Weighted Average) [10]. In the United States, the standard is more severe, since NIOSH (National Institute for Occupational Safety & Health, USA) reduced this value in 2016 to 0.05 mg/m^3^ [11]. Manufacturers of granite and stone materials containing quartz are thus obliged to reduce the concentrations of respirable crystalline silica dust and to ensure the safety of workers by finding solutions to reduce the risks of high exposure to quartz dust.

NIOSH and OSHA (Occupational Safety and Health Administration, Washington DC, USA) investigated the effect of silica content in the material during the manufacture of marble and granite countertops [12]. This research found that in a marble workshop, workers were exposed to silica dust levels of 39 to 45 μg/m^3^ while dry grinding green marble containing 1.8% quartz. In the granite workshop with granites containing at least 10% quartz, exposures to crystalline silica dust suspended in the air were significantly higher, ranging from 89 to 460 μg/m^3^ under the same conditions. It is then reasonable to consider that performing similar operations with materials with a high silica content, such as certain manufactured stone products, could result in higher exposures. A study on exposure to crystalline silica was conducted by Philips and Johnson [13] covering 47 countertop manufacturing shops in three Oklahoma metropolitan areas (USA). Among these shops, 74% reported using primarily dry methods in at least one stage of the countertop manufacturing process, and only 9% reported using dust collection and suppression systems. The sampling results of this study indicated that workers exposed to crystalline silica dust during the manufacture of granite and quartz-based engineered stone countertops were at high risk of exposure to respirable quartz above the permissible exposure limit, even if dry operations were used only to a limited extent. Simcox et al. [14] indicated that the amount of dust emitted when grinding the edges of granite countertops will depend in part on the amount of suction produced by the vacuum system used. They also demonstrated that wet cutting of the stone blocks would most likely reduce dust levels compared to dry cutting. Songmene et al. [15] studied the effect of minimal quantity lubrication (MQL). They found that using water in MQL was effective in reducing the concentrations of FPs generated during the surface polishing of white granite by 20% to 90% depending on the cutting conditions (tool spindle speed, lubrication flow rate). On the other hand, many factors can influence the amount of dust that may be generated and the levels that will result from a particular process, such as the velocity and the manner in which water was applied [16] and the polishing process itself (surface or edge polishing).

Edge-finishing of granite workpieces was initiated by Bahri et al. [17], who investigated the edge shaping of black and white granite samples with a sequence of passes using abrasive tools with increasing grit sizes and a 10 mm radius concave round shape. It was shown that increasing grit size decreases fine particle emissions but increases the generation of ultra fine particles. As the tools must be used in sequence to obtain a good surface finish at the end of the process, particle emission would not be controlled by the tool grit size. They found also that dust emission was influenced by the spindle speed and feed rate and that the best combination for reducing both particle types was 1500 rpm and 1000 mm/min.

The main objective of the present study is to examine the influence of tool shape as well as the variation of lubrication flow and other machining parameters on the generation of fine particles while maintaining a good part surface finish. This will not only help to improve air quality in granite manufacturing workshops and protect workers from high concentrations of particles in their working environment, but the production of good quality parts will also help industrial companies to ensure a certain competitiveness and satisfaction of their customers’ requirements. Only white granite will be used in this study, as it is a material rich in crystalline silica and contains more than 40% of quartz [15]. Experimental tests based on a Design of Experiments approach (DOE) will be carried out using tools with different grit sizes and providing a chamfered and concave shape to the polished edge of the workpiece. The parameters varied in the experimental design are cutting speed, feed rate and the MQL flow rate.

## 2. Experimental Procedure and Methods

### 2.1. Method Description

A 3^3^ factorial Design of Experiments (DOE) was performed varying the polishing conditions (spindle speed, feed rate and MQL lubrication flow) during edge finishing of white granite using tools with concave and chamfer shapes in each polishing phase (roughing G150 and finishing G600). The selection of these parameters was based on the limitations of the MQL lubrication system and the tools used. In fact, the maximum lubrication flow rate that we were able to obtain was 60 mL/min, so an increase in steps of 20 mL/min was elaborated in order to obtain a midpoint at 40 mL/min. As for the rotation speed and the feed rate, the tool manufacturer recommends the use of certain values for each tool, so the choice was made so that the same speeds can be applied to all the tools, while maintaining the midpoint. Table 1 and Table 2 describe the input/output parameters of the white granite edge finishing process studied.

For each (tool-shape/grit-size) configuration, a complete factorial design varying the 3 parameters (N, $V_{f}$, and $Q_{w}$) was carried out with 3 repetitions for each run, giving a total number of 81 tests per configuration as shown in Equation (1). The runs were partially randomized following the variation of the MQL flow rate, since the adjustment of this parameter was manual and too delicate to be carried out.

(1) $$3\times 3^{3}=81\;tests$$

The general form of the model used that expresses the response studied will consider the effect of each parameter in linear and quadratic form, as well as their interactions, as shown in Equation (2).

(2) $$Y_{i}=a_{0}+\overset{3}{\underset{i=1}{\sum }}a_{i}X_{i}+\overset{3}{\underset{i=1}{\sum }}a_{ii}X_{i}^{2}+\overset{3}{\underset{i=1}{\sum }}\overset{3}{\underset{j=i+1}{\sum }}a_{ij}X_{i}X_{j}+\varepsilon$$

where

$Y_{i}$: Response (Total number fine particle concentration Cn_FP, Roughness Ra),

$X_{i}$, $X_{j}$: Factors (N, $V_{f}$, $Q_{w}$),

$a_{0}:$ Arithmetic mean of the response data,

$a_{i}$: Linear effect of the factor i,

$a_{ii}$: Quadratic effect of the factor i,

$a_{ij}$: Interactions effect between factors,

ε: Error.

### 2.2. Experimental Setup

The edge-finishing of granite pieces was carried out on a 3-axis CNC machine (Huron K2 × 10, Huron Graffenstaden SAS, Eschau, France) in which, a Minimum Quantity Lubrication (MQL) flow control system was set up to generate the desired lubrication flow in each run. The cutting fluid used was Oemeta Coolant, Utah, USA. The MQL technique consists in providing lubrication in very small quantities on the targeted areas (tool/part contact). The lubrication is done by the projection of micro-droplets from the cooling hoses which have two output nozzles: one nozzle for the passage of the lubricant, and another nozzle for the passage of the compressed air. The MQL system used has four output hoses, through which the desired flow rate was adjusted, as follows:

1. For 20 mL/min flow rate: Two hoses were used, each one set at 10 mL/min,
2. For 40 mL/min flow rate: Four hoses were used, each one set at 10 mL/min,
3. For 60 mL/min flow rate: Four hoses were used, each one set at 15 mL/min.

The workpiece used was a square of white granite measuring 200 × 200 × 30 mm^3^ with a density of 2667 kg/m^3^ provided by the company A. Lacroix Granit, Saint-Sébastien-de-Frontenac, QC, Canada. This sample was the same as that used in the previous studies [15,17]. It is characterized by medium to coarse grains with an automorphic granular and porphyritic texture, and a quartz percentage of 41% observed by SEM imaging. It should be mentioned that white granite is rich in crystalline silica and contains also 33% of plagioclase and 23% of k-feldspar [15].

The shape tools used in this study are a chamfer tool 3 mm × 45° and a concave tool with 3 mm radius as shown in Table 3. The selection of these two shapes from a wide range of choices given by the supplier was based on two reasons: firstly, the high demand for these tools by granite countertop manufacturers makes them widely used in the industry. Secondly, both tools remove a similar amount of material *V_E_* after one pass of the tool with depth of cut *p* = 0.1 mm, which gives the comparative study more relevance. To achieve the final edge shape, a sequence of tools with different grit sizes must be used in order, as demonstrated in a previous study by Bahri et al. 2021 [17]. In this paper, only two grit sizes will be investigated, which are 150 and 600, with respect to the roughing and finishing stages of a grinding/polishing operation. However, the 45 and 300 grit size tools were still used just for surface preparation of the edge before polishing with 150 and 600 grit size, respectively.

**Figure 1:**
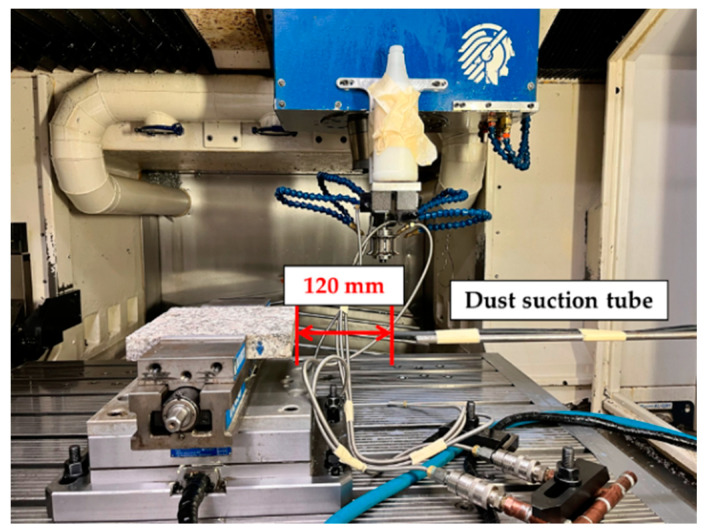
Experimental setup.

The use of an Aerodynamic Particle Sizer (APS) spectrometer (APS, model 3321, TSI Inc., Shore-view, MN, USA) allowed the acquisition of micro-particles with sizes ranging from 0.5 to 20 μm. The data acquisition time was set at 50 s in all the tests performed. The dust samples were pumped through a 10-mm suction tube fixed at a horizontal distance L = 120 mm and a vertical distance H = 0 mm from the tool/workpiece contact area (Figure 1). At the end of the sampling time, the APS equipment provides data consisting of particle concentrations (number, mass and specific surface) as a function of the particle aerodynamic diameters.

The surface roughness was measured with a portable profilometer Surftest SJ-201 from Mitutoyo (Mitutoyo America Corporation, Aurora, IL, USA). This device is equipped with a probe that scans the surface and generates the different roughness parameters (Ra, Rt, Rq, Rz, etc.) of the measured surface. The assembly system of the profilometer on the workpiece holder was designed in-house at ÉTS (École de Technologie Supérieure, Montreal, QC, Canada) to allow direct roughness measurement inside the machine-tool after each run.

### 2.3. Validation Tests

A couple of validation tests were carried out on other white granite samples. These tests will validate the models chosen to represent the FP number concentrations as well as the surface roughness using the chamfered and concave tools and during the two polishing phases. Three white granite samples were used to study the lubrication conditions corresponding to MQL, dry and wet polishing.

New parameter values were tested during the experiments to study the strength of the model. Table 4 shows the configurations used.

## 3. Analysis of Results

### 3.1. Fine Particles Emission

**Figure 2:**
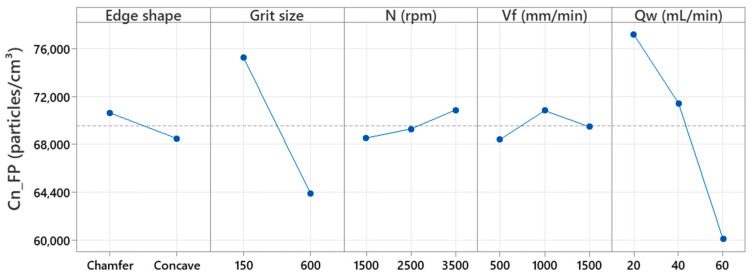
Main effects plot of Cn_FP.

The processing of all the data obtained from the experimentation on the granite sample with the different tools allowed to generation of a main effects plot as displayed in Figure 2, which shows, on average, the effect of each variable and its relation to the response.

We can see from this figure that the grit size and the flow rate are the most significant factors, in that increasing them reduces the total number concentration of FPs. The use of rough tool (Grit 150) produces more fine particles compared to the use of a finishing tool (Grit 600 for example). In finishing operations, more ultrafine particles are expected than fine particles. In the roughing phase where brittle fracture is the material removal mechanism, the tool attacks a hard surface with many ridges and aims to remove as much material as possible to shape the edge of the workpiece; this results in a high concentration of particles. As for the finishing phase, where the material removal mechanism is fluid flow characterized by an increase in tool/part contact pressure and a decrease in the friction coefficient, as pointed out by Saidi et al. [18], the tool does not remove a large amount of material, which explains the low concentration of FPs, since the tool just applies a rubbing force on the edge which gives a low roughness and a good gloss to the polished surface. Increasing the MQL lubrication flow results in minimal FP emissions. Regarding the edge shape, it was found that the emission of FPs was higher using the chamfer tool compared with the concave shaped tool. This result was explained by the fact that the chamfer tool removes a larger volume of material according to Table 3. According to Figure 2, the high cutting speeds and feed rates favored the generation of more FPs during edge-finishing.

**Figure 3:**
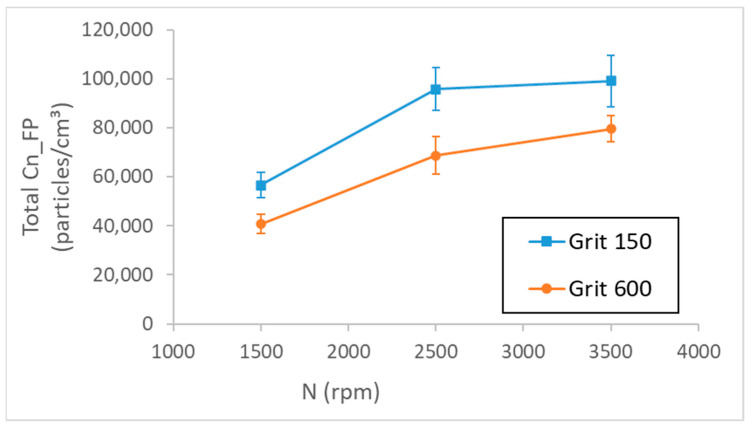
Total number concentration of FPs as a function of spindle speed N while roughing and finishing a concave edge of white granite ($V_{f}\;$= 1000 mm/min, $Q_{w}\;$= 20 mL/min).

Figure 3 shows the effect of spindle speed and grit size on FP emission during edge finishing of white granite with concave shape. Firstly, FPs generation during the roughing phase with grit 150 was significantly higher than that during the finishing phase using grit 600. Secondly, increasing the cutting speed N during both phases of edge-finishing increases total number concentrations of FPs. Increasing the spindle speed from 1500 rpm to 2500 rpm and from 1500 rpm to 3500 rpm increased the FP concentration by approximately 70% and 87%, respectively, for both grit sizes at feed rate $V_{f}\;$= 1000 mm/min and flow rate $Q_{w}$ = 20 mL/min.

**Figure 4:**
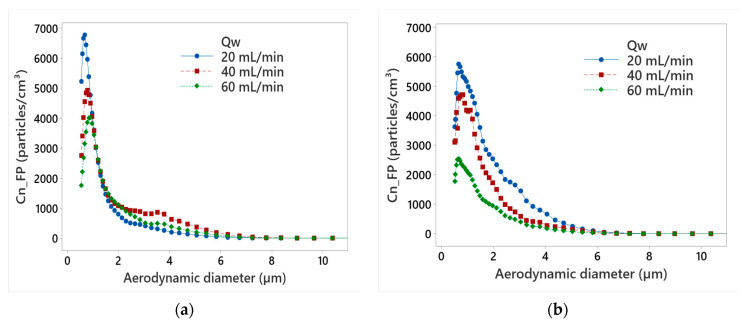
Particle size distribution of Cn_FP at different MQL flow rates using grit size G150 while edge finishing with: (**a**) chamfer shape tool, (**b**) concave shape tool (N = 1500 rpm, $V_{f}\;$ = 1500 mm/min).

Figure 4 illustrates the number concentration of FPs as a function of aerodynamic diameter of the particles for different values of MQL flow rate during the roughing phase using chamfer and concave tools. The chamfer tool generated more FPs than the concave-shaped tool at all lubrication flow rates as already demonstrated in Figure 2. It was also found that increasing the MQL flow rate decreased FP emissions. By using 60 mL/min flow rate, the peak number concentration of FPs is reduced by 45% and 56%, compared to the 20 mL/min MQL flow rate, using chamfer and concave-shaped tools, respectively. These maximum concentrations were observed at aerodynamic diameters below 2 μm, which pose a threat to workers’ safety.

**Figure 5:**
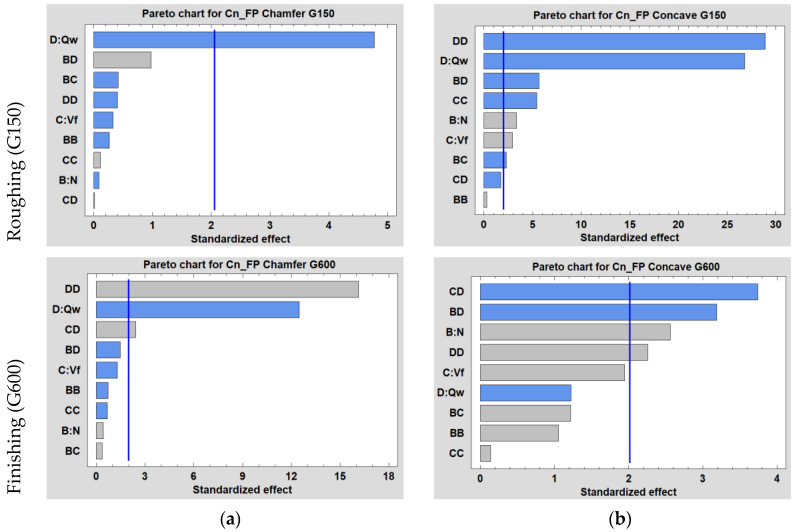
Pareto charts for Cn_FP emitted while edge finishing the white granite workpiece using 150 and 600 grit sizes of: (**a**) chamfer shape tool, (**b**) concave shape tool.

ANOVA analysis is carried out at a 5% significance level (*p*-value below 0.05) and a 95% confidence level. Pareto charts for the different tools/grit sizes used are illustrated in Figure 5 showing the influence of parameters N, $V_{f}$ and $Q_{w}$ selected in the DOE. They compare, in descending order, the statistical significance of the main factors and their interactions. We can see in all graphs that the lubrication flow $Q_{w}$ is the most significant parameter either in linear or quadratic form, or in interaction with another factor.

ANOVA and particle emission regression analyses were performed for both tools during each polishing phase. As the sequence of tools with increasing grit sizes is mandatory during edge finishing, the study of the effect of cutting parameters will obviously depend on the tool used (shape and grit size).

The results of the analysis of variance shown in Table 5 demonstrate that the effects of cutting parameters N, $V_{f}$ and $Q_{w}$ depend on the polishing phase (roughing or finishing) and the shape of the tool used (chamfer or concave).

In Table 5 (a), only the lubrication flow rate $Q_{w}$ in linear form had a significant effect on the generation of FP in number concentration, with a *p*-value less than 0.05 corresponding to a 95% Confidence Interval (CI). The best empirical model we could come up with to predict FP concentrations for Chamfer/G150 represented only 38% of the data (R^2^ = 38%). Therefore, this model was not validated because of its low correlation coefficient.

For the concave shape tool and during the roughing phase (G150), it can be seen from Table 5 (b) that all DOE parameters had a significant effect on FP generation. The most important effect is given by the highest F-ratio value, which is the one for lubrication flow in quadratic form ($Q_{w}^{2}$), followed by the same parameter but in linear form ($Q_{w}$). All significant parameters found in linear, quadratic and interaction form were used to generate the empirical model, which represents 86% of the FP number concentration data in Equation (3).

According to Table 5 (c), while edge finishing with the chamfer tool, a major significance was observed in the effect of the MQL flow in quadratic form ($Q_{w}^{2}$), followed by its linear form ($Q_{w}$) and then its interaction with the feed rate ($V_{f\;}\times Q_{w}$). These parameters were used in the linear regression to obtain an empirical model, shown in Equation (4), that represents well the FP emissions with a correlation coefficient of R^2^ = 92%.

While finishing with the concave shape tool, the cutting speed had a significant effect in linear form (N), in addition to the interaction of $N\times Q_{w}$ and $V_{f}\times Q_{w}$ and the flow in quadratic form ($Q_{w}^{2}$) shown in Table 5 (d). However, these parameters allowed for the generation of a model that represents only 50% of the FP number concentration data. For this reason, this model was not validated.

(3) $$\ln\left(\mathrm{Cn}\_\mathrm{FP}\right)=8.5+3.2\times 10^{-4}N+1.5\times 10^{-3}V_{f}-5.77\times 10^{-7}V_{f}^{2}+0.11Q_{w}-1.5\times 10^{-3}Q_{w}^{2}\;-1.14\times 10^{-7}N\times V_{f}-4.3\times 10^{-6}N\times Qw$$

(4) $$\ln\left(\mathrm{Cn}\_\mathrm{FP}\right)=13.02-0.1\;Q_{w}+1.1\times 10^{-3}\;Q_{w}^{2}+3.8\times 10^{-6}\;V_{f}\times Q_{w}$$

**Figure 6:**
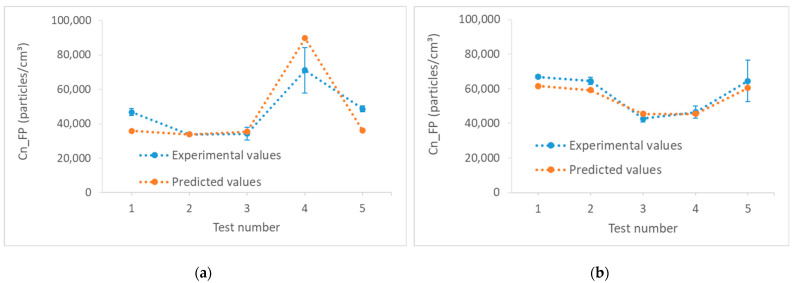
Validation graphs for Cn_FP models: (**a**) Concave using G150, (**b**) Chamfer using G600.

Figure 6 presents the graph of FP number concentrations that were obtained during the validation tests, versus the graph of the values corresponding to the same parameters in the different models developed. We can see that these models are effective and the experimental values approach the predicted values. These models had good correlation coefficients exceeding 80% with relatively small errors (between 10 and 30% for Concave G150, and less than 10% for Chamfer G600). Model errors were calculated as shown in equations 5 and 6, with $e_{i}=y_{i}-x_{i}$, considering that $y_{i}$ is the model prediction, $x_{i}$ is the experimental measurement and $y_{ref}$ was chosen as the maximum value:

Table 6 summarizes the relative errors of the models validated by the experimental tests, where NRMS represents the Normalized Root Mean Square error and MAPE represents the Mean Absolute Percentage Error.

(5) $$NRMS=\frac{RMS}{y_{ref}}=\frac{\sqrt{\frac{1}{n}\sum_{i=1}^{n}e_{i}^{2}}}{y_{max}}$$

(6) $$MAPE=\frac{1}{n}\overset{n}{\underset{i=1}{\sum }}\left|\frac{e_{i}}{x_{i}}\right|\times 100\;\%$$

### 3.2. Surface Finishing

**Figure 7:**
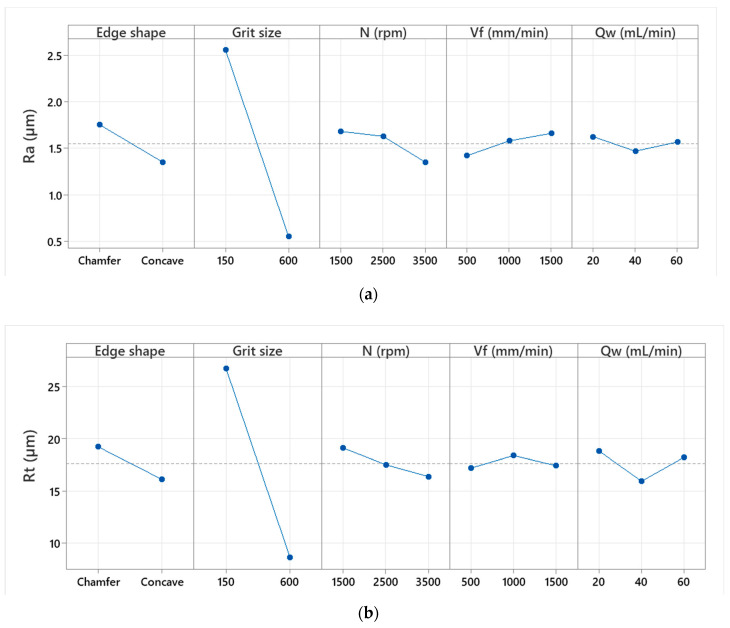
Main effects plot of roughness: (**a**) Ra, (**b**) Rt.

The same approach used to analyze the particle concentrations was employed to study the surface finish. In Figure 7, main effects plots of arithmetic roughness Ra and Rt are shown, giving a general overview of the behavior of studied responses towards the variations in experimental design parameters such as edge shape, grit size, spindle speed, feed rate and lubrication rate. The plots were generated using Minitab software. The details of the use of the software are elaborated upon in the Discussion section.

**Figure 8:**
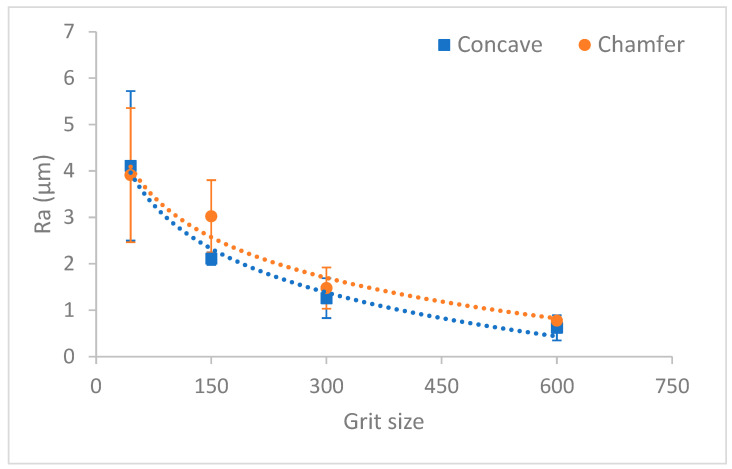
Roughness Ra depending on grit size for chamfered and concave shape tools.

**Figure 9:**
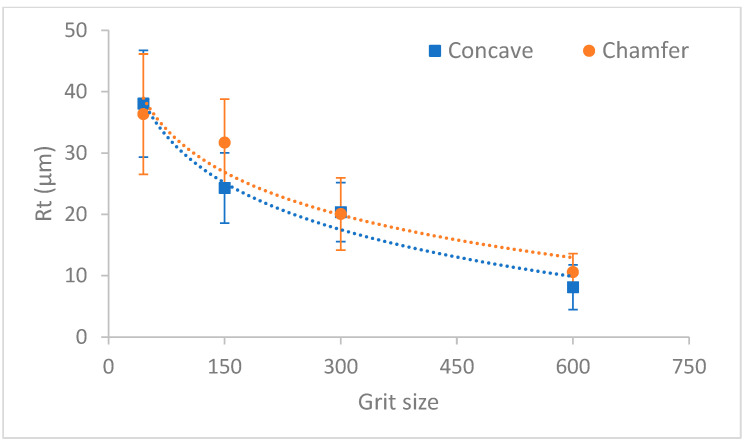
Roughness Rt depending on grit size for chamfered and concave shape tools.

**Figure 10:**
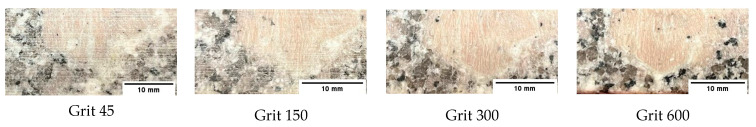
Effect of tool grit size on polished white granite surface using concave shape tool.

It can be quickly seen that the pattern of the curves for all factors is the same for both Ra and Rt responses. Grit size had the largest significant effect among all other factors. It is obvious that the transition from roughing (polishing with grit 150) to finishing phase (polishing with grit 600) improves the surface finish more regardless the cutting conditions used. This is confirmed with others results presented in Figure 8, Figure 9 and Figure 10. In terms of edge shape, a concave tool shape seems to give better surface finish, thus a lower Ra and Rt roughness, than a chamfered shape. Rotational and feed rates need to go through ANOVA analysis to determine their statistical significance. However, the lubrication rate had no remarkable effect on roughness when examining all the data at once. This could vary depending on the polishing phase in question.

The variation of roughness values Ra and Rt as a function of grit size for the two edge shape tools are shown in Figure 8 and Figure 9, respectively. Based on these graphs, the surface finish of the concave shape tool was slightly better than the chamfer shape tool throughout the edge finishing process. This difference is probably due to the diamond distribution in each tool of different shape, but this interpretation requires more in-depth studies for validation. It should be noted here that the more diamonds on a tool surface, the better is the roughness of the polished part using said surface.

The equations for the tendency graphs representing the roughness parameters Ra and Rt as a function of grit size for each edge shaping tool are mentioned in Table 7. These equations modeling the output variable had high correlation coefficients (>90%), which will predict, depending on the tool used (grit size and shape), the roughness values Ra and Rt that may be obtained.

The effect of grit size is observed through the evaluation of the surface profiles of edges made with different tools (chamfer and concave). The polishing conditions were set at N = 2500 rpm, $V_{f\;}$= 1000 mm/min and $Q_{w}\;$= 60 mL/min. Comparisons between the edge surface roughness in the two cases were carried out using the arithmetic mean deviation Ra in addition to the observation of surface profiles as shown in Table 8. It can be noted that there are several dips and peaks in the surface profiles defined by high negative and positive amplitudes as well as non-periodic amplitudes while scanning the surface of the edge. This is due to the heterogeneity of the granite material, which is composed of several crystals stuck together. Therefore, the choice of the comparison criteria for the surface roughness was made for an arithmetic mean given by the mean deviation Ra. On a same scale, the amplitudes tend to decrease significantly from one grit size to the next with both chamfered and concave shaped tools. This explains the effect of an increased amount of diamond present in tools with larger grit sizes, which can minimize the irregularities and ridges present in the material and improve its surface quality. Figure 10 shows an example of polished surface with the different grit sizes of concave edge shape at N = 2500 rpm, $V_{f}\;$= 1000 mm/min and $Q_{w}\;$= 60 mL/min.

The images of the polished granites shown in Figure 10 were taken using the camera of an iPhone 13 (Dual 12 MP (mega pixels) camera system: Wide and Ultra Wide cameras; Wide: ƒ/1.6 aperture and Ultra Wide: ƒ/2.4 aperture and 120° field of view). The scale bar was added using Image J 1.53 k software (National Institutes of Health, USA) by measuring a reference distance on the granite edge with a ruler and introduce it in the software as a known distance so it generates the right scale bar.

**Figure 11:**
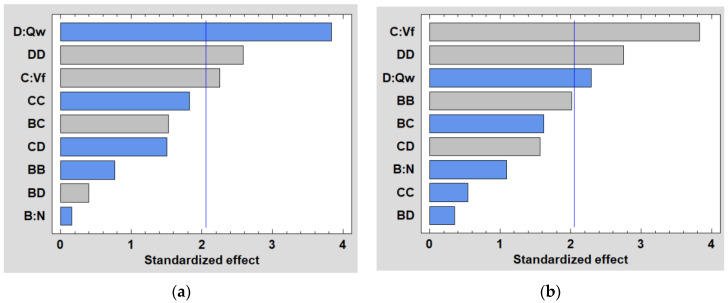
Pareto charts for Ra while edge-finishing using: (**a**) chamfer tool, (**b**) concave tool.

The main importance in edge-finishing from a manufacturing point of view is given to the surface quality that the finishing tool gives. Therefore, the statistical study will focus only on the cutting parameters used in the finishing phase. The results of ANOVA analysis show that while the statistically significant effects on the Ra response were the same for both edge finishing tools, the order of significance, however, differed between the two. The Pareto charts in Figure 11 show that the lubrication rate $Q_{w}$ appears to be the most dominant factor when using the chamfered tool, but it is almost insignificant for the concave shape tool. In addition, the feed rate $V_{f}$ is the most important factor when using the concave shaped tool, whereas it has a minimal importance with the chamfered tool.

Unfortunately, the best empirical models developed for roughness as a function of cutting parameters (N, $V_{f}$ and $Q_{w}$) had low correlation coefficients R^2^, less than 50%. Thus, these models were not validated.

**Figure 12:**
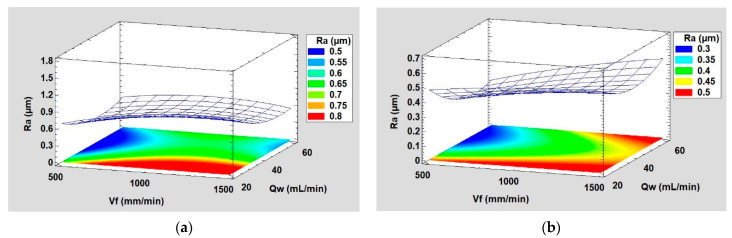
3D response surface plots of Ra when using: (**a**) chamfer shape tool, (**b**) concave shape tool (Grit 600, N = 2500 rpm).

The response surface method (RSM) was employed to determine the evolution of the roughness parameter Ra in the finishing phase using both chamfer and concave shape tools. The 3D plots generated are presented in Figure 12. These plots show the variation of the roughness Ra as a function of the two parameters feed speed ‘$V_{f}$’ and lubrication flow ‘$Q_{w}$’ at a cutting speed N = 2500 rpm. The areas in which the roughness Ra was minimal are identified in the 3D graphs by the blue color, while large roughness values are determined by the red color. Therefore, it can be said that the low roughness values Ra, a sign of a good surface finish, are obtained mainly with low feed rates and high lubrication rates. This applies to both edge shaping tools. Indeed, a combination of a low feed rate with a high enough lubrication flow allows the tool to polish the edge surface in good conditions that avoid its wear and heating, considering that severe conditions on the tool are indicative of a bad surface finish in all machining and polishing processes.

## 4. Discussion

**Figure 13:**
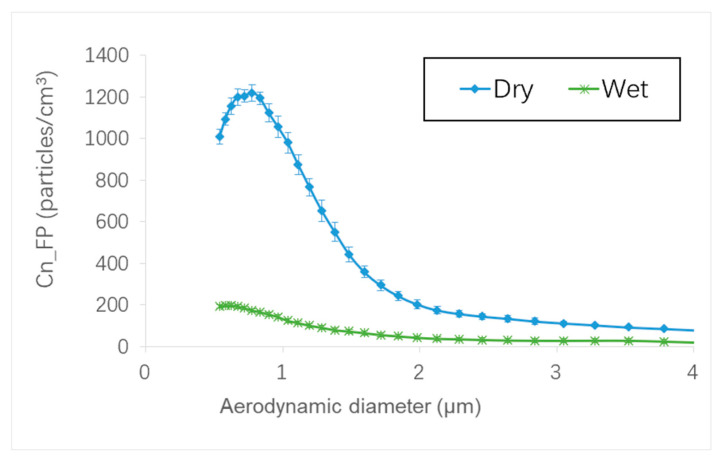
Particle size distribution of FP number concentration with different lubrication modes (Chamfered tool, Grit 150, N = 2500 rpm, $V_{f}$ = 1000 mm/min).

Among the salient points that can be noted in regard to the generation of FP is that the effect of the polishing parameters depends primarily on the edge shape to be realized and the polishing phase (grit size tool used). The parameter that had the greatest effect on decreasing FP generation was the grit size of the tools used, as demonstrated by Bahri et al. [17] and Saidi et al. [18]. However, it should be noted that the transition through the different tools with their different grit sizes is necessary to achieve the finished edge. Therefore, it is important to be aware that this parameter is one of the most significant in FP generation and, although the operators cannot control it, they will be able to avoid, for example, the realization of several roughing passes that would generate much more FP compared to the finishing passes. Regarding the edge shape to be realized, the use of the concave shape tool is preferable since it generates not only less FP in concentration than the chamfered shape tool, but also provides a better surface finish throughout the edge finishing process. The lubrication flow rate was the second most important factor after the grit size, playing a dominant role in FP emission. The lower the lubrication rate during the polishing pass, the higher the FP generation. During the validation tests, extreme lubrication conditions such as dry and wet polishing were also studied in addition to the MQL. Figure 13 clearly shows that the use of wet lubrication seems to be the best solution for decreasing FP emissions with a maximum concentration equal to 198 particles/cm^3^, compared to 1220 particles/cm^3^ for dry polishing under the same conditions. Therefore, polishing with full lubrication reduces the maximum number concentration of FPs corresponding to particles smaller than 1 µm diameter by about 85% as compared to dry condition.

**Figure 14:**
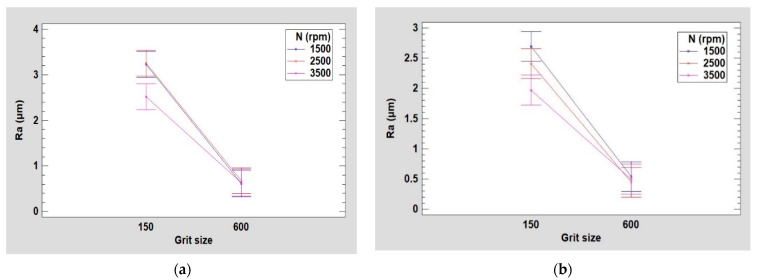
Variation in roughness Ra as a function of grit size for different spindle speeds using: (**a**) chamfer shape tool, (**b**) concave shape tool.

The study of edge surface roughness which aims to find out the parameters that influence the response parameter Ra during the edge finishing of white granite revealed that the greatest importance on the surface finish during the whole process is the grit size of the tool used. It is obvious that the increase in grit size during the polishing process will result in a better surface finish, which is why this study covered the roughing and the finishing phases. Varying the spindle speed did not contribute towards obtaining a better surface finish in the finishing phase (grit 600). In the roughing phase (grit 150), however, its increase led to a decrease in Ra (Figure 14). This result confirms the work of Bahri et al. [17], which gave due importance to the effect of spindle speed on the generation of particles during the roughing phase, since in the end, the roughness desired by the customer will be attained progressively when reaching the finishing stage.

**Figure 15:**
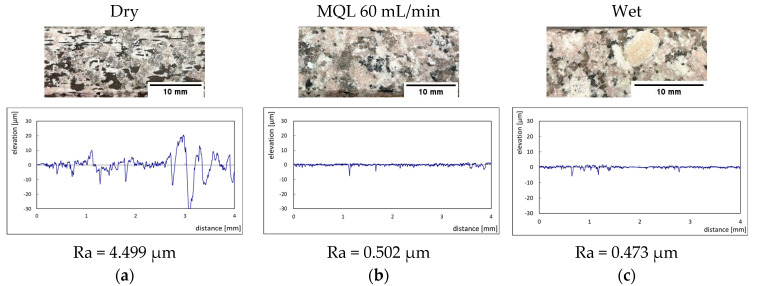
Surface roughness polished: (**a**) dry, (**b**) with MQL, and (**c**) wet (Chamfer shape, Grit 600, N = 2500 rpm, $V_{f}$ = 1000 mm/min).

The MQL flow rate as well as the feed rate were among the most influential parameters for the surface finish during the finishing phase. The difference between dry and lubricated polishing can be observed in the images taken using the camera of an iPhone 13 (Dual 12 MP (mega pixels) camera system: Wide and Ultra Wide cameras; Wide: ƒ/1.6 aperture and Ultra Wide: ƒ/2.4 aperture and 120° field of view) in Figure 15, where the dry polished surface shows black spots resulting from the burning caused by sparks during the passage of the tool without any lubrication, as well as a roughness value Ra almost 10 times greater than that obtained in the presence of lubricant, i.e., MQL at a flow rate of 60 mL/min, or in wet lubrication. This observation is in keeping with what has been demonstrated in the work of Bahri et al. [17]. As mentioned previously in the case of Figure 10, the scale bar seen in each image in Figure 15 was inserted using the software Image J 1.53 k (National Institutes of Health, USA), whereby a measured distance along the granite edge was entered as input into the software, which then generated the correct scale bar for the image.

As the main objective of this study was the optimization of the edge-finishing process, desirability functions were used on Minitab software through the generated models. These functions aim to:

1. Decrease the number concentration of FP during the roughing phase, since roughness is not important in this phase,
2. Obtain the best surface finish, without forgetting to minimize as much as possible the emission of particles during the finishing phase.

Table 9 and Table 10 summarize the desirability function parameters used for the optimization of cutting conditions (N, $V_{f}$ and $Q_{w}$) in the roughing and finishing phases, respectively, when grinding white granite edge with both tools (chamfer and concave). The importance parameter is maintained at 1 in the roughing phase since it concerns only the optimization of a single response which is the Cn_FP, but in the finishing phase it will be judicious to pay more attention to the roughness variable Ra as the most important parameter for the manufacturer (60%), even before the number concentration of FP (40%). The results of this optimization analysis are shown in Table 11 indicating the optimal values to be used with the chamfer and the concave shape tools when polishing the white granite edge in roughing and finishing phases.

## 5. Conclusions

Grinding parameters examined in this study influence the responses such as FP concentration and surface roughness. A good combination of polishing parameters, depending on the tool shape used and its grit size, could minimize the number of particles generated and improve the surface finish of the polished edge. Based on the experiments carried out in this investigation, the following conclusions may be drawn.

1. Edge finishing using the concave shape tool is better than the chamfer shape tool as both the generation of FP is less and the surface finish is better during the entire edge finishing process.
2. The use of lubricant is essential during grinding of granite edges. In the absence of lubrication, a large quantity of dust smaller than 1 µm is generated, in addition to a poor surface finish characterized by black burn spots and giving a Ra roughness 10 times greater than that obtained with wet lubrication or with a high MQL flow. Polishing with flood lubrication reduces the maximum number concentration of FPs corresponding to particles smaller than 1 µm diameter by about 85% as compared to dry polishing.
3. The use of lubricant in the maximum tested MQL mode (60 mL/min) produced a surface finish with Ra-value of 0.502 μm, comparable to that obtained when using flood lubrication (0.473 μm). In dry condition the obtained Ra-value was 4.99 μm.
4. The optimization carried out has allowed us to identify, depending on the tool shape used and the polishing phase, the parameters that minimize FP generation while ensuring a good surface finish. While these parameters do not necessarily form an ideal combination, they certainly meet the objectives of this study.

## Figures and Tables

**Table 1:** Input parameters of white granite edge finishing process.

Factor	Levels					
Edge shape	Chamfer 3 mm × 45°			Concave R3 mm		
Tool grit size	150			600		
Spindle speed N (rpm)	1500		2500		3500	
Feed rate $V_{f}$ (mm/min)	500		1000		1500	
Lubrication flow rate $Q_{w}$ (mL/min)	20		40		60	

**Table 2:** Response variables of white granite edge finishing process.

Response Variable		Description
FP emissions	Cn_FP (particles/cm^3^)	Number concentration of FPs
Roughness	Ra (μm)	Arithmetic mean deviation of the surface profile
	Rt (μm)	Total height of the surface profile

**Table 3:** Edge tools used and volume of material removed during one pass of edge-finishing.

Tool grit size		Chamfer	Concave
150 (roughing)		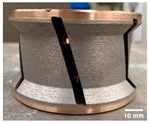	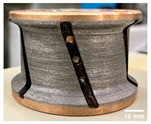
600 (finishing)		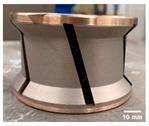	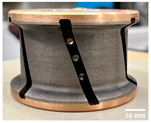
Volume of material removed per pass	V_E_	7.5 mm^3^	6.5 mm^3^

**Table 4:** Sequence of validation tests and configurations used.

Edge Shape	Grit Size	Test Number	N (rpm)	$V_{f}$ (mm/min)	$Q_{w}$ (mL/min)
Concave	150	1	1500	1500	60
		2	3000	1250	60
		3	1000	750	60
		4	2000	1500	40
		5	2500	1000	60
Chamfer	600	1	3000	1250	60
		2	1000	750	60
		3	2500	500	40
		4	2000	1500	40
		5	2500	1000	60

**Table 5:** ANOVA tables of Cn_FP depending on each tool shape and grit size.

**(a) ANOVA of Cn_FP for Chamfer/G150**					
**Source**	**SS**	**DF**	**MS**	**F-Ratio**	***p*-Value**
*N*	$2.14\times 10^{5}$	1	$2.14\times 10^{5}$	0.01	0.932
$V_{f}$	$3.06\times 10^{6}$	1	$3.06\times 10^{6}$	0.11	0.747
$Q_{w}$	$6.58\times 10^{8}$	1	$6.58\times 10^{8}$	22.78	0.000 *
*N* ×*N*	$2.01\times 10^{6}$	1	$2.01\times 10^{6}$	0.07	0.794
$N\times V_{f}$	$5.04\times 10^{6}$	1	$5.04\times 10^{6}$	0.17	0.679
$N\times Q_{w}$	$2.75\times 10^{7}$	1	$2.75\times 10^{7}$	0.95	0.338
$V_{f}\times V_{f}$	$3.8\times 10^{5}$	1	$3.8\times 10^{5}$	0.01	0.909
$V_{f}\times Q_{w}$	$6.66\times 10^{3}$	1	$6.66\times 10^{3}$	0.00	0.988
$Q_{w}\times Q_{w}$	$4.64\times 10^{6}$	1	$4.64\times 10^{6}$	0.16	0.691
Error	$7.5\times 10^{8}$	26	$2.88\times 10^{7}$		
Total	$1.86\times 10^{9}$	52			
**(b) ANOVA of Cn_FP for Concave/G150**					
*N*	$2.7\times 10^{8}$	1	$2.7\times 10^{8}$	11.36	0.002 *
$V_{f}$	$2.04\times 10^{8}$	1	$2.04\times 10^{8}$	8.58	0.007 *
$Q_{w}$	$1.71\times 10^{10}$	1	$1.71\times 10^{10}$	718.47	0.000 *
N×N	$2.38\times 10^{6}$	1	$2.38\times 10^{6}$	0.1	0.754
$N\times V_{f}$	$1.27\times 10^{8}$	1	$1.27\times 10^{8}$	5.33	0.029 *
$N\times Q_{w}$	$7.65\times 10^{8}$	1	$7.65\times 10^{8}$	32.23	0.000 *
$V_{f}\times V_{f}$	$6.96\times 10^{8}$	1	$6.96\times 10^{8}$	29.33	0.000 *
$V_{f}\times Q_{w}$	$7.21\times 10^{7}$	1	$7.21\times 10^{7}$	3.04	0.093
$Q_{w}\times Q_{w}$	$1.98\times 10^{10}$	1	$1.98\times 10^{10}$	835.7	0.000 *
Error	$6.17\times 10^{8}$	26	$2.37\times 10^{7}$		
Total	$4.67\times 10^{10}$	52			
**(c) ANOVA of Cn_FP for Chamfer/G600**					
*N*	$7.02\times 10^{6}$	1	$7.02\times 10^{6}$	0.2	0.653
$V_{f}$	$5.87\times 10^{7}$	1	$5.87\times 10^{7}$	1.71	0.197
$Q_{w}$	$5.36\times 10^{9}$	1	$5.36\times 10^{9}$	156.02	0.000 *
N×N	$1.9\times 10^{7}$	1	$1.9\times 10^{7}$	0.55	0.46
$N\times V_{f}$	$4.68\times 10^{6}$	1	$4.68\times 10^{6}$	0.14	0.713
$N\times Q_{w}$	$7.67\times 10^{7}$	1	$7.67\times 10^{7}$	2.23	0.142
$V_{f}\times V_{f}$	$1.63\times 10^{7}$	1		0.48	0.494
$V_{f}\times Q_{w}$	$2.05\times 10^{8}$	1	$2.05\times 10^{8}$	5.98	0.018 *
$Q_{w}\times Q_{w}$		1	$8.94\times 10^{9}$	260.41	0.000 *
Error	$1.51\times 10^{9}$	44	$3.43\times 10^{7}$		
Total	$1.62\times 10^{10}$	53			
**(d) ANOVA of Cn_FP for Concave/G600**					
*N*		1	$5.08\times 10^{8}$	6.55	0.014 *
$V_{f}$	$2.93\times 10^{8}$	1	$2.93\times 10^{8}$	3.77	0.056
$Q_{w}$	$1.15\times 10^{8}$	1	$1.15\times 10^{8}$	1.49	0.229
N×N	$8.58\times 10^{7}$	1	$8.58\times 10^{7}$	1.11	0.298
$N\times V_{f}$	$1.15\times 10^{8}$	1	$1.15\times 10^{8}$	1.48	0.230
$N\times Q_{w}$	$7.88\times 10^{8}$	1	$7.88\times 10^{8}$	10.14	0.002 *
$V_{f}\times V_{f}$	$1.54\times 10^{6}$	1	$1.54\times 10^{6}$	0.02	0.888
$V_{f}\times Q_{w}$	$7.21\times 10^{7}$	1	$7.21\times 10^{7}$	14.01	0.000 *
$Q_{w}\times Q_{w}$	$1.08\times 10^{9}$	1	$1.08\times 10^{9}$	5.09	0.029 *
Error	$3.95\times 10^{8}$	44	$7.76\times 10^{7}$		
Total	$6.8\times 10^{9}$	53			

* Significant factor.

**Table 6:** Error values for validation of Cn_FP models.

	NRMS	MAPE	Ideal Value
Cn_FP for Concave G150	12.43%	15.83%	<10%
Cn_FP for Chamfer G600	6.54%	6.18%	

**Table 7:** Tendency equations of Ra and Rt depending on grit size for chamfered and concave shapes.

Edge Shape Tool	Tendency Equation (N = 3500 rpm; $V_{f}$ = 1500 mm/min; $Q_{w}$ = 20 mL/min)	R^2^
Chamfer	Ra =−1.26ln(G)+8.9	95%
	Rt =−10.05ln(G)+77.2	91%
Concave	Ra =−1.36ln(G)+9.15	98%
	Rt =−11.01ln(G)+80.3	97%

**Table 8:** Evolution of surface profiles and roughness Ra of white granite edge (chamfer and concave) as a function of grit sizes.

	Chamfer	Concave
Grit 45	Ra = 6.631 µm	Ra = 5.241 µm
	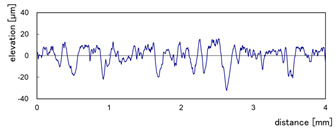	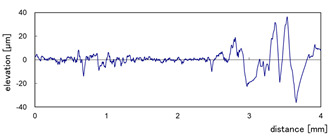
Grit 150	Ra = 1.829 µm	Ra = 2.558 µm
	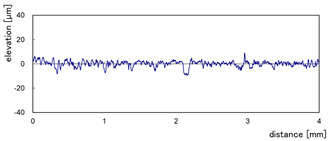	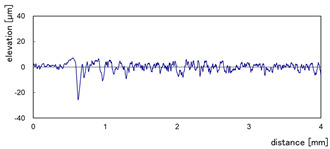
Grit 300	Ra = 1.516 µm	Ra = 0.894 µm
	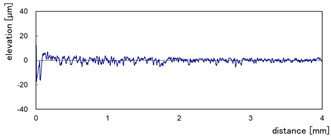	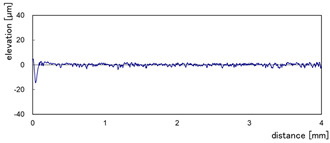
Grit 600	Ra = 0.502 µm	Ra = 0.507 µm
	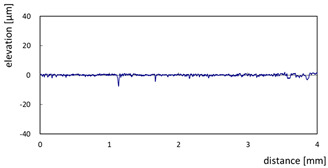	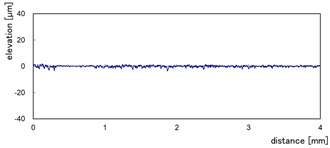

**Table 9:** Summary of desirability function parameters for chamfer and concave shape tools in the roughing phase.

Response	Goal	Edge Shape	Target	Lower	Upper	Importance
Cn_FP	Minimize	Chamfer	61,100	61,100	94,600	1
		Concave	20,000	20,000	121,000	

**Table 10:** Summary of desirability function parameters for chamfer and concave shape tools in the finishing phase.

Response	Goal	Edge Shape	Target	Lower	Upper	Importance
Cn_FP	Minimize	Chamfer	40,500	40,500	102,000	0.4
Ra			0.3	0.3	2	0.6
Cn_FP		Concave	37,100	37,100	90,200	0.4
Ra			0.2	0.2	1	0.6

**Table 11:** Optimal values for polishing white granite edge.

Tool Shape	Roughing Phase (Grit 150)			Finishing Phase (Grit 600)		
	N (rpm)	$V_{f}$ (mm/min)	$Q_{w}$ (mL/min)	N (rpm)	$V_{f}$ (mm/min)	$Q_{w}$ (mL/min)
Chamfer	1500	1500	60	3500	500	40
Concave	3500	1500	60	2328	500	40

## Data Availability

Data available on request due to restrictions.
